# Integrating Metabolomics and Network Pharmacology to Explore the Protective Effect of Ginsenoside Re against Radiotherapy Injury in Mice

**DOI:** 10.1155/2022/5436979

**Published:** 2022-03-11

**Authors:** Chunmiao Yu, Jiaqi Fu, Lidong Guo, Miaomiao Yu, Donghua Yu

**Affiliations:** Heilongjiang University of Chinese Medicine, Harbin 150040, China

## Abstract

Ionizing radiation (IR) can cause radiation damage, mutagenesis, or carcinogenesis in the irradiated subject. It is manifested as metabolic disorders of the body and damage to the immune system, nervous system, and endocrine system, which can lead to physiological and pathological changes and endogenous metabolic disorders. Ginsenoside Re (G-Re), a single component of traditional Chinese medicine, has a certain ameliorating effect on radiation damage. However, its mechanism of action in the treatment of radiotherapy injury remains unclear. With this purpose, the hematopoietic function of mice damaged by X-ray radiation was studied, and the protective effect of G-Re on mice damaged by radiation was preliminarily evaluated. Network pharmacology and metabolomics analysis are used to further reveal the mechanism of G-Re to improve radiation damage through metabolomics research. Results of metabolomics analysis showed that 16 potential biomarkers were identified as participating in the therapeutic effect of G-Re on IR. Most of these metabolites are adjusted to recover after G-Re treatment. The pathways involved included glycerophospholipid metabolism, sphingolipid metabolism, and linoleic acid metabolism. According to network pharmacology analysis, we found 10 hub genes, which is partly consistent with the findings of metabolomics. Further comprehensive analysis focused on 4 key targets, including SRC, EGFR, AKT1, and MAPK8, and their related core metabolites and pathways. This study combines metabolomics and network pharmacology analysis to explore the key targets and mechanisms of G-Re in the treatment of IR, in order to provide new strategies for clinical treatment of radiotherapy injury.

## 1. Introduction

Ionizing radiation (IR) refers to the general term for radiation that can cause ionization of affecting substances, including X-rays, charged particles of *α* and *β*, and uncharged neutrons. It has the characteristics of high energy, high frequency, and short wavelength [1]. It has brought huge economic and social benefits to human modern life and also has the potential harm to the human body. It has grown up to be a kind of “invisible bomb” in life, causing serious harm to human life and health at any time [2, 3]. The application of IR in medicine including diagnosis and treatment, especially the continuous update of X-knife, CT instrument, and gamma-knife treatment methods, has become an important method and has been more and more widely used. Radiation can not only kill tumor cells but also cause damage and death of normal cells and cause local and systemic adverse reactions in patients [4]. Hematopoietic system damage caused by radiotherapy is the most common, which is mainly manifested as bone marrow suppression, significantly increased risk of anemia and bleeding, decreased immune function, and enhanced complications.

Total body irradiation (TBI) will provoke a continuous increase in the level of ROS in the irradiated body, leading to chronic oxidative stress [5]. When ionizing radiation acts on organisms, the impact on the hematopoietic system is mainly manifested in the inhibition and destruction of bone marrow hematopoietic stem cells and hematopoietic stromal cells, as well as the impact on peripheral blood cells. Hematopoietic stem cells protect the hematopoietic system from exhaustion under various stress conditions under steady-state conditions; when the body is exposed to radiation, hematopoietic stem cells and their self-renewal ability are impaired, resulting in decreased blood cell and platelet counts, decreased hematopoietic function, and hematopoietic system development. Long-term or permanent damage and bone marrow failure and body death may occur [6].

Hematopoietic dysfunction is the main clinical symptom of radiotherapy injury. In recent years, many studies have found that antiradiation effects are found in Chinese herbal medicines that invigorate qi and blood and enhance immunity, and its active ingredients have become an important way to develop radiopharmaceuticals. In addition, traditional Chinese medicine holds the characteristics of the high source, small toxicity, and low price, so traditional Chinese medicine antiradiation active ingredients have become a research hotspot in recent years. *Panax ginseng* C. A. Meyer was first published in Herbal Lection. It is the rhizome of a perennial herbaceous plant belonging to Araliaceae and *Panax ginseng*. It has the elegant names of God grass, goblin, and jade essence and is known as the king of herbs [7]. With the development of modern medical technology, the pharmacological effects and application value of ginseng have been gradually confirmed by modern pharmacology and clinical medicine, and it has been widely used in various preparations of decoctions and Chinese patent medicines, which has established its special position in Chinese medicine in my country [8]. Ginsenoside Re (G-Re) is the main active ingredient of ginseng. G-Re has a good protective effect on UVB-induced skin damage, and its mechanism of action is linked to the scavenging of free radicals and antioxidant effects [9]. In addition, G-Re can also play a role in resisting radiation damage by protecting the immune system and cardiovascular system, resisting oxidative stress, inhibiting cell apoptosis, and reducing lipid peroxidation [10, 11].

Metabolomics is the study of various metabolic changes that occur in an organism under the action of physiology, pathology, or drugs. By measuring the changes of endogenous metabolites in the organism, the target can be more easily discovered, thereby revealing its mechanism of action [12]. The serum is a commonly used biological fluid that is easily available and rich in information in metabolomics. Monitoring the level of specific metabolites in serum has become an important method for detecting early disease. Serum metabolite profiles can be considered as an important indicator to monitor the physiological and pathological status of organisms. Compared with other biofluid analyses, serum metabolite profiles have many advantages, contribute to the understanding of the mechanism of disease occurrence and development at the metabolic level, and provide information that can identify early and differential metabolic markers of disease [13, 14]. Radiation injury is a pathological state caused by ionizing radiation, which can result in a series of changes in metabolites. Many of these studies are based on the search for biomarkers related to radiation dose in metabolites that can be used for early diagnosis and early warning of radiation damage. The development of network pharmacology is based on the increasing knowledge of the interaction between proteins and molecules, which is of great help in understanding the pathogenesis of TCM syndromes and the treatment mechanism of TCM [15, 16]. Therefore, combining traditional Chinese medicine with metabolomics provides an effective way to scientifically explain the metabolic mechanism of traditional Chinese medicine as an auxiliary drug for radiotherapy.

At present, there are few reports on the radiation resistance of ginseng and the protective mechanism of ginsenosides against X-ray radiation damage and the mechanism of action from the aspect of metabonomics is still unclear. Potential therapeutic targets were screened based on metabonomics and network pharmacology to prove the therapeutic effect of G-RE on ionizing radiation injury. A mouse model of radiation injury induced by X-ray was established and serum metabolomics was used to screen differential metabolites. Potential targets for G-RE treatment of IR were identified by network pharmacology, and a comprehensive network of metabolomics and network pharmacology was constructed. Using a combination of metabonomics and network pharmacology, this study provides a strategy to understand the underlying mechanisms of radiation damage. At the same time, G-Re is considered to be an auxiliary drug for exogenous radiotherapy to study its radiation protection effect on organisms and its mechanism.

## 2. Materials and Methods

### 2.1. Reagents

Ginsenoside Re (Re purity) were purchased from Nanjing Chunqiu Biological Engineering Co., Ltd., and identified according to the Chinese Pharmacopoeia (2015 Edition). Perchloric acid, glacial acetic acid, anhydrous calcium chloride, and formaldehyde were obtained from Shanghai Macklin Biochemical Co., Ltd. (China). Leu-enkephalin was obtained from Sigma-Aldrich company (USA). Acetonitrile was obtained from Thermo Fisher Scientific company (USA). HPLC-grade formic acid and methanol were obtained from Dikma Technologies company (CA). The assay kits used to determine IL-2, IL-6, IL-12, and IgM were purchased from Jiancheng Bioengineering Institute (Nanjing, China).

### 2.2. Animal and Experimental Design

Thirty Kunming mice (Weighing approximately 20 ± 2 g) were supplied by the Liaoning Changsheng Biotechnology Co., Ltd., (Liaoning, China) with the license number SCXK (Liao) 2020-0001. All mice were housed in an animal breeding room under specific pathogen-free (SPF) conditions with free access to fodder and purified water at a temperature (22 ± 2°C) with 50–60% humidity and under a 12 h light/dark cycle. All animal procedures were performed in accordance with the Guide for Care and Use of Laboratory Animals and the related ethical regulations of Heilongjiang University of Chinese Medicine (process number: 2018052325).

After acclimatization, thirty KM mice were divided into three groups as follows: normal control group (*n* = 10), radiation model group (*n* = 10), and dosage group of G-Re (240 mg/kg/d, *n* = 10). The G-Re dose was chosen based on the daily intake from our previous study of Chinese. The control and model groups were administered corresponding volumes of normal saline; the G-Re group was orally administrated with G-Re. Drugs were administered for 20 days by gavage. 9th day after the intragastric administration, except for the control group, the model group and the G-Re group were subjected to whole-body one-time irradiation with X rays (irradiation dose of 5.0 Gy). After irradiation, the G-Re group continued to gavage the G-Re, and the control group and the model group continued to give normal saline.

### 2.3. Sample Collection and Preparation

#### 2.3.1. Serum Collection and Detection of Biochemistry Parameters

After the last intragastric administration of G-Re for 24 hours, blood samples were collected from the orbit and then divided into two aliquots. Serum was obtained from one aliquot by centrifugation at 3500 rpm for 15 min after the blood samples clotted and stored at −80°C.

Serum samples were taken to measure the IL-2, IL-6, IL-12, and IgM indicators according to the kit instructions. Methanol (400 mL) was added to serum (100 *μ*L) and vortexed for 1 min followed by centrifuged at 12,000 rpm for 15 min, and the obtained supernatant was then transferred to another tube and the supernatant was filtered through a 45 *μ*L filter and moved into a microinlet tube. The obtained supernatant was set aside until use for the UPLC-MS analysis.

#### 2.3.2. Collection and Biochemical Test of Mouse Spleen and Bone Marrow

The spleen was taken out and weighed, put in the preconfigured Bouin's solution for 24 hours, and then took out. The number of nodules protruding from the surface of the spleen (CFU-S) was counted by naked eye or a magnifying glass. Get out the mice femur and use scissors to subtract the joints at the upper and lower ends of the femur. Use a syringe to draw 10 mL of 5 mmol/L CaCl_2_ solution to slowly flush the bone marrow into the centrifuge tube until the inner wall of the femur is white and place the centrifuge tube with bone marrow under 4°C for 30 min. After centrifugation at 3500 r/min for 10 min, discard the supernatant, then add 5 mL 0.2 mol/L HClO_4_ solution to the precipitate and mix thoroughly, then cool the resulting solution to room temperature in a water bath at 90°C for 15 min, and centrifuge at 3500 r/min for 15 min. Discard the precipitate and aspirate the supernatant, and use a UV spectrophotometer to measure the absorbance of the obtained supernatant at 268 nm to calculate the DNA content of the bone marrow.

(1) $$\begin{array}{c} \text{DNA}\left(\mu \text{g}\right)=40\times 50\times A\left(268\text{nm}\right). \end{array}$$

### 2.4. Chromatographic and Mass Spectrometric Conditions

In our experiment, chromatographic separation was performed on a UPLC BEH C18 column (100 mm × 2.1 mm, 1.7 *μ*m i.d.; Waters Corporation, Milford, Massachusetts, USA) using a Waters ACQUITY UPLC System (Waters Corporation). A 3 *μ*L aliquot of the sample was injected into the column, which was maintained at 35°C. The UPLC mobile phase consisted of 0.1% formic acid acetonitrile (solvent A) and water (solvent B). The gradient duration was 9 min at a constant flow rate of 0.4 mL/min. The metabolites were eluted using a linear gradient of 99–80% B for 0.5 min, 80–40% B for 0.5–2.5 min, 40–34% B for 2.5–4 min, 34–20% B for 4–5 min, 20–14% B for 5–7.5 min, 14–1% B for 7.5–8 min, and 1% B for 8–9 min. The column eluent was directed to the mass spectrometer for analysis. A blank was analyzed between every five samples to wash the column.

Mass spectroscopy was performed by a Waters Micromass Q-TOF (Quadrupole Time-of-Flight) Micromass Spectrometer (Waters Corporation) connected to electrospray ionization (ESI) operating in positive and negative modes and a full scan mode from m/z 100 to 1500 for 0 to 16 min. The desolvation gas rate of nitrogen was set to 750 L/h, the desolvation temperature was 350°C, the cone gas rate was set at 20 L/h, and the source temperature was 110°C. The capillary voltage was set at 1.3 kV and 1.5 kV in positive ion mode and in negative ion mode, respectively. The cone voltage was set at 60 V in positive and 70 V in negative ion mode. For accurate mass acquisition, a lock mass of leucine enkephalin was used through a lock spray interface at a flow rate of 40 *μ*L/min. The lock spray frequency was set at 0.48 s, and the lock mass data were averaged over 10 scans for correction.

### 2.5. Data Processing and Analysis

The UPLC/MS data were processed using the Micromass MarkerLynx Application Version 4.1 (Waters, Milford, MA, USA) and Progenesis QI software, which allowed data alignment, peak matching, deconvolution, and reduction to yield a table of mass and retention time pairs with the associated intensities for all detected peaks. Mathematical treatments of data such as principal component analysis (PCA) and partial least squares discriminant analysis (PLS-DA) apply a statistically driven model in order to determine latent variables indicative of hidden relationships between the observed data. All ions to be analyzed were arranged in descending order according to variable importance in the project (VIP) values and S-plot. Variables with VIP >1.0 and *q* value <0.05 were considered to be important in discriminating biomarkers between different groups. The databases used to confirm the pick-out potential biomarkers were the Human Metabolome Database (HMDB: http://www.hmdb.ca/spectra/ms/search) and the Kyoto Encyclopedia of Genes and Genomes (KEGG: http://www.genome.jp/kegg/). MetaboAnalyst (http://www.metaboanalyst.ca/) and other metabolic pathway databases carry out enrichment analysis and network construction of related metabolic pathways.

Data collected in biochemical assay were expressed as mean ± SD. Statistical analyses were performed using SPSS 21.0 software. Comparisons between groups were measured by two-sided test. *P* values < 0.05 were considered statistically significant.

### 2.6. Network Pharmacology Analysis

In order to better reveal the key metabolites and related proteins, by constructing a metabolite-protein pathway network, Cytoscape 3.7.2 (Cytoscape Consortium, CA, USA) is used for network construction. The chemical structural formula, SMILE standard structural formula, and PubChem CID of ginsenoside Re were searched through the PubChem database (https://pubchem.ncbi.nlm.nih.gov/). Then import SwissTargetPrediction database (http://swisstargetprediction.ch/) and PharmMapper database (http://59.78.98.102/pharmmapper/get.php) to screen related targets. “Radiation” is used as keywords in GeneCards (https://www.genecards.org/), OMIM database (https://www.omim.org/), and DisGeNET (https://www.disgenet.org/). The intersection of disease target and drug target is used as the prediction target of drug action on disease. The Uniprot database (http://www.uniprot.org/) was used to standardize the names of genes and proteins. The target information of interaction between G-Re and radiation damage obtained above was imported into STRING database (https://string-db.org/) to obtain the correlation between potential targets. Cytoscape V3.7.2 software was used to visually analyze the results of protein interaction analysis in STRING database and construct protein-protein interaction network. Fourth, using the David (http://david.nifcrf.gov/) database, the obtained intersecting genes were subjected to a GO annotation analysis (gene-ontology) and a KEGG pathway analysis (Kyoto Encyclopedia of Genes and Genomes). Differential metabolites identified in metabolomics were introduced into Cytoscape with Metscape to obtain a compound-reaction-enzyme-gene network. This structure was carried out to visualize the interactions between metabolites, pathways, enzymes, and genes. Key metabolites and proteins are then identified by combining the compound-reaction-enzyme-gene network with central genes and metabolic pathways.

## 3. Results

### 3.1. The Levels of Pharmacodynamic Index in the Mice Serum

Colony forming unit-spleen (CFU-S) are the remaining hematopoietic stem cells with the ability to proliferate and divide after exposure to ionizing radiation [17]. They proliferate and differentiate in the spleen and finally form a certain number and size of splenic colonies, forming a rounded shape visible to the naked eye. Each splenic nodule is referred to as a splenic nodule forming unit. CFU-S and the changes in bone marrow DNA together represent the body's ability to restore hematopoietic tissue. As shown in Table 1, the spleen surface of the mice in the normal control group was smooth and no splenic nodules were formed. CFU-S gradually formed on the surface of the spleen after irradiation. Compared with the model, CFU-S increased in the G-Re group and the difference was statistically significant (*P* < 0.05). As shown in Table 1, compared with the normal group, the bone marrow DNA content of the model group was significantly reduced (*P* < 0.01), indicating that radiation would cause the bone marrow DNA content to decrease, and the damage to the bone marrow would seriously damage the normal function of the body's hematopoietic system. Compared with the model group, the bone marrow DNA content in the G-Re group was significantly greater, and the difference was extremely significant (*P* < 0.01).

Ionizing radiation can affect the production of cytokines, which have played a role in many radiation-related studies and have promoted bone marrow remodeling after radiotherapy and chemotherapy. As shown in Table 2, compared with the normal group, the serum concentrations of IL-2 and IL-12 in the radiation model group were significantly decreased, while the serum concentrations of IL-2 and IL-12 in the G-Re group were significantly increased (*P* < 0.05). After radiation, the serum IL-6 level of mice was detected, and the radiation model group had the highest level, and the difference was significant compared with the normal group (*P* < 0.01). The experimental results showed that compared with the model group, the G-Re group could effectively increase the IL-6 concentration, and the difference was significant (*P* < 0.05). As a glycoprotein of immunoglobulin, IgM can reflect the body's immune response to pathogens in the blood. After the mice were irradiated with X-rays, the concentration of IgM in the serum of irradiated mice was reduced. Among them, mice in the irradiated model group decreased the most, and the difference was statistically significant compared with the normal control group (*P* < 0.05). The results showed that the G-Re can increase the IgM concentration level, and the difference is statistically significant compared with the model group (*P* < 0.05).

### 3.2. Multivariate Statistical Analysis of Serum Metabolic Profiles of Irradiated Mice

**Figure 1:**
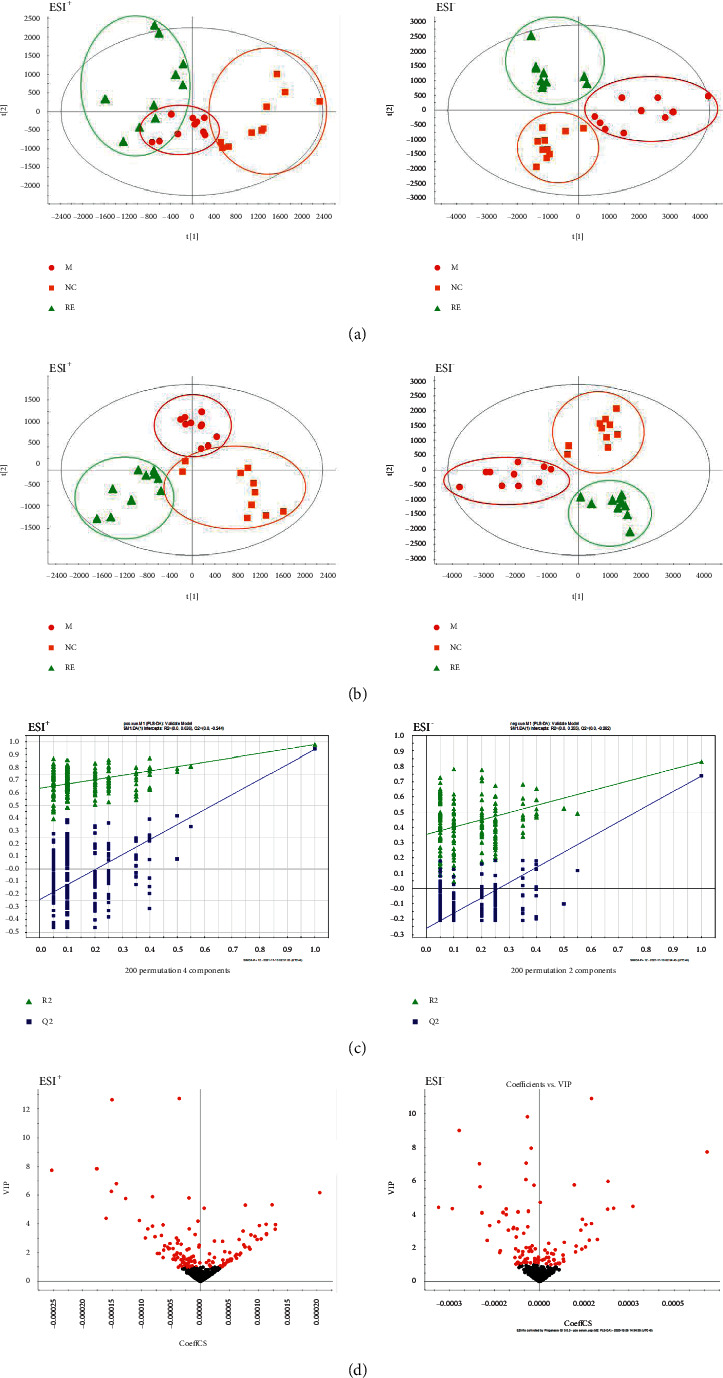
(a) PCA score of mice under positive (ESI+) and negative ion mode (ESI-); (b) PLS-DA analysis under positive (ESI+) and negative ion mode (ESI-); (c) statistical validations obtained by 200X permutation tests under positive (ESI+) and negative ion mode (ESI-); (d) VIP-plot under positive (ESI+) and negative ion mode (ESI-).

Changes of endogenous metabolites in serum of mice were induced by X-ray, and the data were analyzed by combining PCA and PLS-DA to screen the different potential metabolites. As shown in Figure 1(a) in the PCA in positive and negative ion mode, the sample points of the normal group and the model group were obviously separated, indicating that the serum metabolic profile of the mice after radiation injury was obviously disordered. A clear separation between the G-Re treated and model groups can be observed in the PCA score plot where each coordinate represents one sample, with significant differences in metabolites across samples. In order to further verify the separation of each sample in the normal group, the model group, and the ginsenoside dose group and to maximize the separation between groups, the supervised PLS-DA method was adopted, and the model established by this supervised method had a high interpretation rate and prediction rate. As can be shown from Figure 1(b), PLS-DA analysis significantly improved the ability of data separation and aggregation. The serum metabolic profile of the three groups of mice showed their own clustering, which were placed in three separate regions of the scatter score chart. In the PLS-DA diagram of the normal group and the radiation model group, the metabolic trajectories showed good similarity within each group and little crossover and overlap, which were far away from each other, indicating that obvious biological changes occurred in the serum of mice after radiation induction and also indicating that the metabonomics technology could effectively distinguish the difference between normal and radiation injury. As can be seen from the PLS-DA diagram of the normal group, the model group, and the G-Re group, there was a deviation trend between the G-Re group and the radiation model group, the G-Re group was more concentrated, and the intragroup difference was small, and the deviation was closer to the normal group. From the point of view of the hour, G-Re affected the serum metabolic profile of the radiation mice and alleviated the deviation caused by ionizing radiation.

The stability and predictive power of the PLS-DA model should be evaluated by cross-validation (CV) and substitution experiments. As shown in Figure 1(c), R2X = 0.263, R2Y = 0.86, *Q*2 = 0.74 (negative ion mode) and R2X = 0.681, R2Y = 0.971, *Q*2 = 0.925 (positive ion mode), indicating that the PLS-DA model under the two ion modes has a low risk of overfitting and the model has a good predictive ability.

As shown in Figure 1, VIP-plot (D), in the “*V*-shaped distribution of ion fragments,” the closer the fragments to the top of the two sides, the greater the contribution rate to the change of metabolic profile trajectory, and red is the selected biomarker. Therefore, VIP >1 was selected as the criteria for screening potential biomarkers. The preliminary screened potential metabolites were tested by SPSS 21.0 software for *t*-test, and the variables without significant difference (*P* < 0.05) were excluded. HMDB, KEGG, and other databases were used to determine the structural information of the screened potential markers, which were imported into MetaboAnalyst 5.0 for online analysis of metabolic pathways. The corresponding pathways were analyzed and elaborated, and the corresponding metabolic pathways involved in the potential markers were sorted out.

### 3.3. Identification of Potential Biomarkers and Pathway Analysis

**Figure 2:**
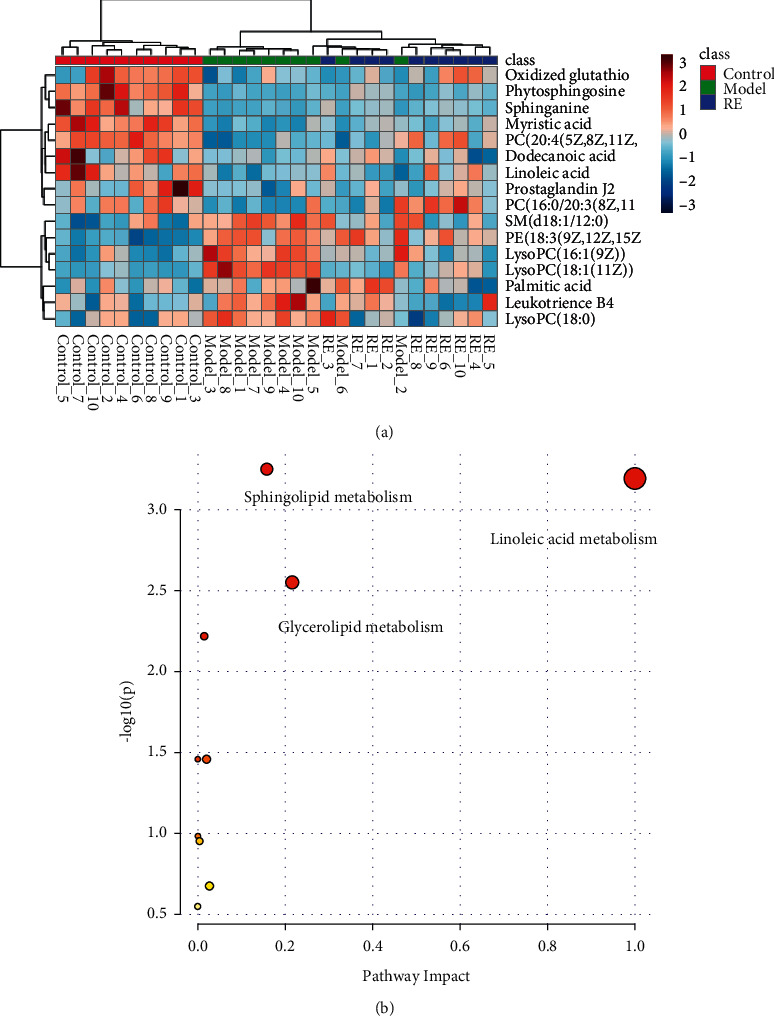
Heat map of cluster analysis of mouse serum metabolites and metabolic pathway analysis diagram.

Through the search for QI software and HMDB and KEGG network database, 16 common and significantly different metabolites were screened as the characteristic biomarkers of radiated mice before and after intervention, as shown in Table 3. In the heat map of markers obtained by hierarchical clustering analysis, the horizontal axis represents different experimental groups, and the vertical axis represents the biomarkers compared in groups. Each row represents the expression levels of different markers in different samples and all markers in each sample. The color blocks in different positions represent the relative expression of the corresponding position markers. The changes in the content of 16 potential biomarkers in the serum of mice between each group can be directly seen from the heat map (Figure 2(a)). Potential biomarker content could be clearly distinguished between the normal group and the radiation model group, and the potential biomarker level could be clearly distinguished between the G-Re dose group and the model group. The level of potential markers in the G-Re dose group moved closer to the normal control group than the model group.

Import the relevant 16 differential metabolite ion information table in the irradiated mouse serum into MetaboAnalyst 5.0 Pathway Analysis for topological analysis and set the critical value of the metabolic pathway impact value to 0.10, and the pathways higher than this value will be as a potential target path. As shown in Figure 2(b) and Table 4, serum metabolic pathways in radiation-damaged mice were mainly related to linoleic acid metabolism, sphingolipid metabolism, and glycerophospholipid metabolism. Under the intervention of G-Re, the content of metabolites was adjusted back, and the disorder of related metabolic pathways was improved. From the metabolic level, it was verified that G-Re had a better recovery effect on mice damaged by ionizing radiation.

### 3.4. Network Pharmacology Analysis

**Figure 3:**
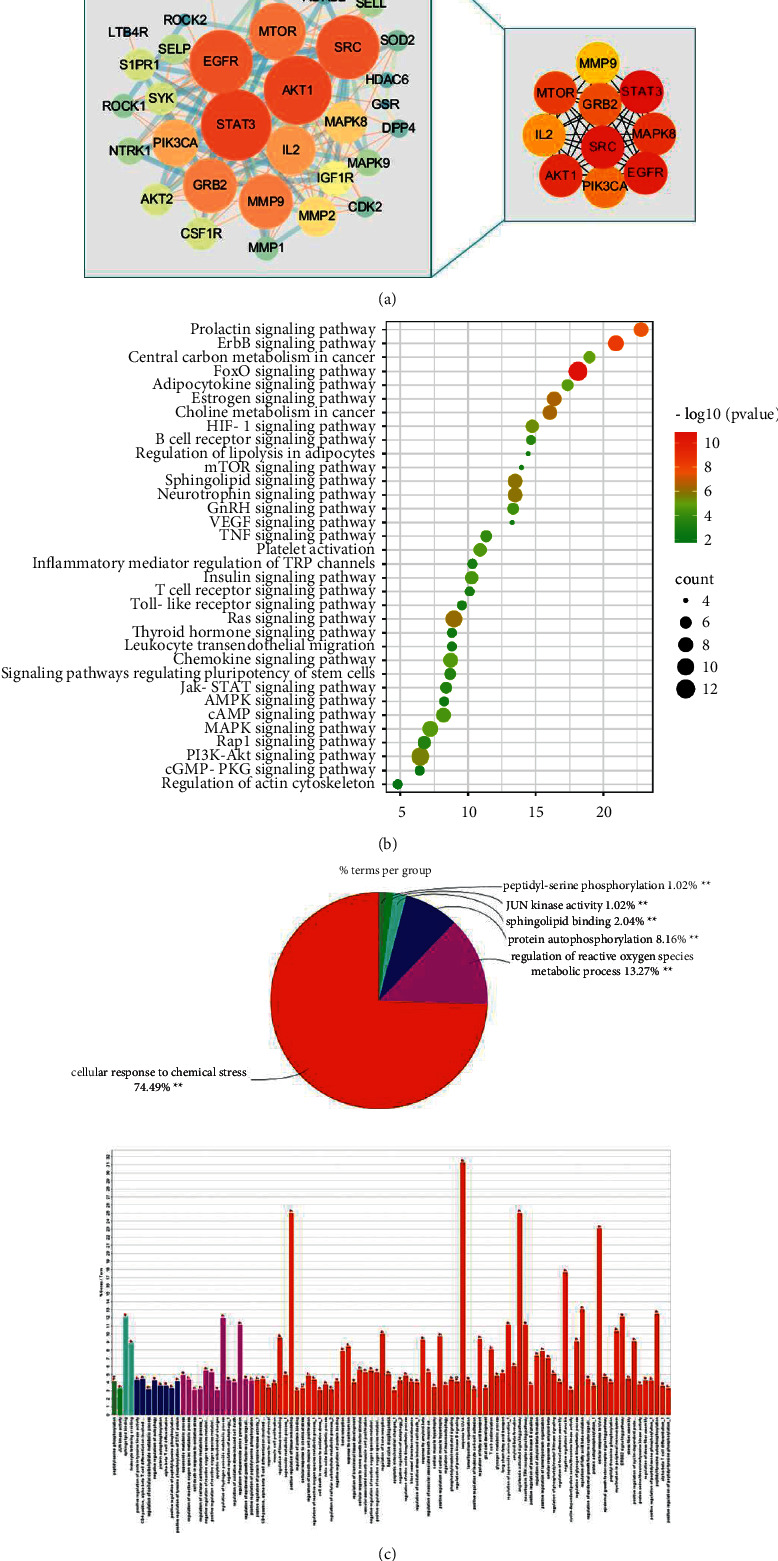
Network pharmacology analysis of Ginsenoside Re in the treatment of IR. (a) PPI network diagram of RE treating IR. Node color reflects its degree; (b) diagram of the results from the KEGG pathway enrichment analysis (*P* ≤ 0.05); (c) pie chart and bar chart of the results from the GO enrichment analysis (*P* ≤ 0.05).

In order to further explore the mechanism of G-Re on IR, we conducted a network pharmacology study. The intersection of disease-related genes and drug active ingredient-related targets was obtained, and 34 genes were obtained (Table 5). As shown in Figure 3(a), in order to identify the hub genes of G-Re and HUA, Cytoscape 3.8.2 was used for visual analysis to construct the protein-protein interaction network. At the same time, cytoHubba plug-in was used to screen the core targets, and the top 10 genes were considered as the hub genes combined with the scores of the calculation method (STAT3, SRC, EGFR, AKT1, MTOR, MAPK8, GRB2, PIK3CA, IL-2, and MMP9). GO annotation and KEGG pathway enrichment analysis of the obtained Re anti-IR potential target genes by David (Figures 3(b) and 3(c)). GO enrichment analysis showed mainly cellular response to chemical stress, peptidyl-serine phosphorylation, JUN kinase activity, sphingolipid blinding, protein autophosphorylation, and regulation of reactive oxygen species metabolic process. According to the KEGG enrichment analysis, the significantly affected pathways were FoxO signaling pathway, sphingolipid signaling pathway, PI3K-Akt signaling pathway, MAPK signaling pathway, chemokine signaling pathway, TNF signaling pathway, B cell receptor signaling pathway, and adipocytokine signaling pathway.

### 3.5. Comprehensive Analysis of Metabolomics and Network Pharmacology

**Figure 4:**
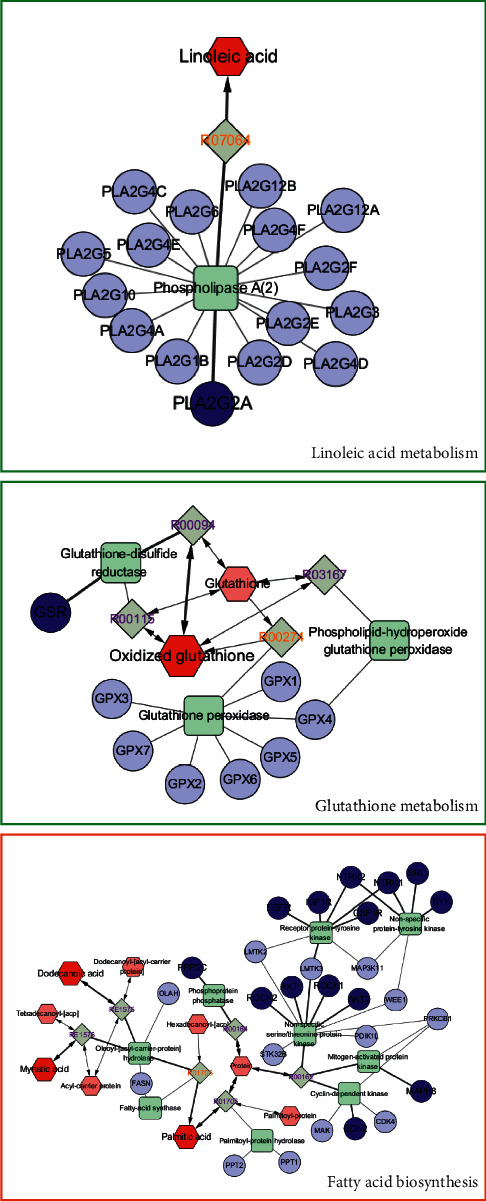
Compound-reaction-enzyme-gene network of key metabolites and targets. Red hexagons, gray diamonds, green round rectangles, and purple circles represent active compounds, reactions, proteins, and genes, respectively.

To comprehensively understand the mechanism of G-RE against IR, we constructed an interaction network based on metabolomics and network pharmacology. The differential metabolites were imported into the MetScape plug-in of Cytoscape, and the compound-reaction-enzyme-gene network was collected (Figure 4). By matching potential targets identified in network pharmacology with genes in MetScape analysis, we found GSR and PLA2G2 are related to oxidized glutathione and linoleic acid, and PPP5, ROCK1, ROCK2, AKT1, AKT2, CDK2, MAPK8, EGFR, IGF1R, CSF1R, NTRK2, NTRK1, SRC, and SYK are related to palmitic acid, dodecanoic acid, and myristic acid (Table 6). They may play an important role in the therapeutic effect of G-Re on IR. Among these genes, SRC, EGFR, AKT1, and MAPK8 are the hub genes.

## 4. Discussion

**Figure 5:**
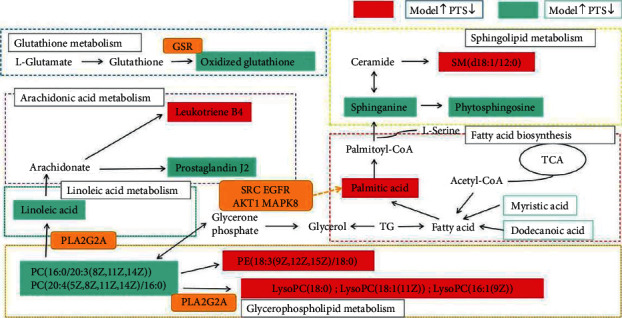
Network of the potential biomarkers associated with IR.

The metabolites of the body change with the pathological changes of the body, and the identification of these changes through metabolomics can provide valuable information about the effects of radiation on health, help to find the potential markers of disease, and provide a new opportunity to elucidate the development mechanism of radiation-induced injury. In our experiment, we discovered 16 kinds of G-Re metabolites to IR and their related pathways. But metabolomics research is limited to listing potential metabolites and related pathways, without further exploring their direct relationship. Network pharmacology can further verify the therapeutic regulation of metabolic networks and promote the identification of key targets and biomarkers. By combining metabolomics and network pharmacology, four hub genes (SRC, EGFR, AKT1, and MAPK8), five key metabolites (linoleic acid, palmitic acid, dodecanoic acid, myristic acid, and oxidized glutathione) and four related pathways (linoleic acid metabolism, fatty acid biosynthesis, and glutathione metabolism) were discovered. This comprehensive strategy found the core target and mechanism and provided a more accurate G-Re counter-IR network (Figure 5).

### 4.1. Linoleic Acid

Linoleic acid is a short-chain polyunsaturated fatty acid, which is an essential nutrient for the human body. It exists in animal fat in the form of glycerol ester together with other fatty acids and is the core component of the plasma membrane of most cells. It can lower cholesterol, soften blood vessels, prevent cardiovascular diseases, and promote lipid metabolism [18]. Phospholipase A2 is an esterase that catalyzes glycerophospholipid to produce fatty acids and lysophospholipids. PLA2G2A, a member of the phospholipase family, is a secretory phospholipase with a molecular weight of only 16 kDa [19]. Most of the currently known functions of PLA2G2A are related to inflammation, immune response, antithrombosis, cell proliferation, ischemic injury, and allergic reactions [20]. Linoleic acid can be constituted by phosphatidylcholines (PC) under the action of PLA2G2A and participate in the body's lipid metabolism.

Our experimental results show that the content of linoleic acid in the model group is significantly lower than that in the control group. It is speculated that IR may activate excess reactive oxygen species, while linoleic acid belongs to unsaturated double bonds containing unsaturated fatty acids and polyunsaturated fatty acids. The double bond is very sensitive to IR and is easily affected by hydrogen peroxide to cause structural changes, damage the cell membrane, and directly affect the immune function of the human body. G-Re can regulate the abnormal linoleic acid metabolism pathway in mice and increase the level of linoleic acid in serum, so as to reduce the cell damage of the body.

### 4.2. Sphingolipid Metabolism

Results of network pharmacology showed that the sphingolipid signaling pathway is included in the KEGG of being reintervened with IR-related genes, which is consistent with the results of metabolomics analysis. Sphingolipid metabolism is a complex reaction of sphingosine-based compounds in animal and plant membranes, especially in nerve cell membranes or in the rich myelin sheath of brain tissues and central nervous system tissues. Sphingolipids provide many bioactive metabolites that regulate cell proliferation, differentiation, and apoptosis, such as ceramide, sphingosine, and 1-phosphate sphingosine, which play an important role in cell signal molecular transduction [21]. To clarify the mechanism of sphingolipid metabolism and its relationship with diseases lays a foundation for clinical treatment. Therefore, the study of sphingolipid metabolism is of great significance for understanding the changes of serum metabolism before and after radiation injury. The differential metabolites mapped in sphingolipid metabolism in this experiment mainly included sphingomyelin (SM (d18 : 1/12 : 0)), sphinganine, and phytosphingosine metabolites. Among them, the metabolites of SM (d18 : 1/12 : 0) changed significantly. Compared with the normal control group, the index of SM (d18 : 1/12 : 0) in the radiation model group increased. Sphingomyelin is composed of sphingosine, phosphocholine, and fatty acid. It can generate ceramide under the action of sphingomyelinase. It is contained in the lipid membrane of the cell and is especially abundant in the membranous myelin sheath of nerve cell axons. This may be explained by the decrease in sphingomyelinase activity after radiation, which leads to the accumulation of sphingomyelin in the blood. However, in the dose group after G-Re treatment, SM (d18 : 1/12 : 0) was significantly downregulated in comparison with the model group. It may be due to the recovery of sphingomyelinase activity after G-Re treatment, which reduces the accumulation of sphingomyelin. As a second messenger, ceramide exists between the cell membrane and cytoplasm and is an essential cell signaling factor related to IR, oxidative stress, and inflammation. It can inhibit the oxidative stress response caused by TNF-alpha and to reduce the release of ROS [22]. Sphinganine is catalyzed by ceramidase to produce ceramides, and then the synthesis of ceramides is blocked due to the lack of sphingolipase, and the content of ceramides decreases, thus affecting the synthesis of sphinganine. Phytosphingosine is a class of lipid compounds with immuno-enhancing activity. It can be used as a precursor of ceramide and can induce cell apoptosis. It was found that the contents of sphinganine and phytosphingosine were significantly decreased in the model group compared with the normal group, while G-Re could significantly regulate the contents of sphinganine and phytosphingosine, thereby further regulating sphingolipid metabolism.

### 4.3. Glycerolipid Metabolism

Metabolites that changed significantly in glycerophospholipid metabolism included phosphatidylcholines (PC) and lysophosphatidylcholines (LysoPC) and phosphatidylethanolamine (PE). Glycerolipid metabolism is involved in the metabolism of serum samples, which are a special hydrolysis reaction existing in the body and an important metabolic pathway in the study of the pathogenesis of many diseases. It can be seen that glycerolipid metabolism is of great significance to the exploration of lipid metabolism in the body [23].

Phosphatidylcholine is the main glycerophospholipid that participates in the metabolism of glycerophospholipids and is also the principal component of cell membrane lipoprotein and lipid bilayer structure. It holds the unique characteristics of polar and nonpolar parts. It can be inserted into its own cell membrane to prevent lipid peroxidation, inhibit the breakage of lipid disulfide bonds, prevent cells from being destroyed and apoptosis, and maintain their cell integrity [23]. Lysophosphatidylcholine in the blood is drawn from the hydrolysis of phosphatidylcholine by phospholipase A2 (PLA2). LysoPC plays its biological function by activating ion channels, inducing cell apoptosis and increasing oxidative stress-induced inflammation and can activate a variety of immune cells. IR affects the activity of phospholipase and causes changes in the content of phosphatidylcholine, which will lead to a large number of lysophosphatidylcholine compounds aggregation inflammation, leading to serious functional disorders of the body and the development of diseases with metabolic abnormalities [25]. PLA2G2A screened by network pharmacology is the coding gene of small molecular weight secreted phospholipase A2IIA, which is associated with the process of PC synthesis of LysoPC. It corresponds to our metabolomics results.

PC (20 : 4 (5Z, 8Z, 11Z, 14Z)/16 : 0) and PC (16 : 0/20 : 3 (8Z, 11Z, 14Z)) belongs to phosphatidylcholine. We found that compared with the normal group, the content of phosphatidylcholine in the radiation model group was reduced, and the decrease in PC content after ionizing radiation may be due to radiation-induced damage to body cells and accelerated metabolism, thereby increasing the absorption of PC in the blood, while the content of the G-Re group was significant upregulation, which may inhibit cell damage caused by IR and protect cell integrity by unregulated phosphatidylcholine. Lysophosphatidylcholines (LysoPC (16 : 1 (9Z)), LysoPC (18 : 1 (11Z)), and LysoPC (18 : 0)) are involved in the metabolism of glycerophospholipids and are monoglycerophospholipids. It is converted from phosphatidylcholine and plays a major role in lipid metabolism, energy metabolism, and oxidative damage to the body. In general, the content of LysoPC in cells or tissues is low under normal conditions, but when the content is too high, it can damage the membrane system of cells. In this experiment, LysoPC (18 : 0), LysoPC (18 : 1 (11Z)), and LysoPC (16 : 1 (9Z)) are all upregulated in the radiation model group, indicating that IR leads to a high content of lysophatidylcholine thus affecting the permeability of vascular endothelial cells. It can also accelerate inflammation of blood vessels, affect platelet aggregation process, and cause hemolysis or necrosis of tissue cells, doing harm to the body. Compared with the model group, the content level after G-Re was significantly decreased. PC generates phosphatidylserine under the catalysis of phosphatidylserine synthase and further obtains phosphatidylethanolamine (PE) by phosphatidylserine decarboxylase [26]. In our experiment, it was found that PE (18 : 3 (9Z, 12Z, 15Z)/18 : 0) content increased after radiation, while G-Re can reduce LysoPC and PE content, increase PC content, regulate the balance of glycerophospholipid metabolism, and achieve the effect of anti-ionizing radiation.

### 4.4. Fatty Acid Biosynthesis

In addition, studies have also found that palmitic acid, myristic acid, and dodecanoic acid are involved in the fatty acid biosynthesis, which are structural components of cell biofilms. IR induces damage to mitochondrial structure and function, aggravates cellular oxidative stress, and results in the deposition of fatty acids in the cytoplasm, resulting in the inability of their products to be effectively utilized or transported, resulting in accumulation and impaired fatty acid metabolism. The experiment showed that the content of palmitic acid increased in the model group, but decreased significantly in the G-Re group. It was speculated that G-Re could also restore lipid metabolism disorder by regulating fatty acid metabolism.

In addition, hub genes (SRC, EGFR, AKT1, and MAPK8) screened by network pharmacology analysis were correlated with palmitic acid. As a core member of SRC family protein tyrosine kinases (SFKs), SRC plays an important role in mitosis, cell growth, and tumorigenesis [27, 28]. Epidermal growth factor receptor (EGFR) is a glycoprotein receptor on the surface of cell membrane, belonging to Erb B receptor family, and a receptor tyrosine kinase (RTK). EGFR has tyrosine kinase activity. When combined with ligand-EGF, EGFR can phosphorylate tyrosine residues, activate EGFR, and promote the growth and proliferation of tumor cells [29, 30]. EGFR-associated antibodies and inhibitors have proved to be useful for tumor therapy in basic oncology studies [31]. AKT1 is a serine/threonine kinase that plays an important role in cell metabolism, cell survival, cell cycle regulation, transcriptional regulation, and other biological processes [32]. Akt can inhibit cell apoptosis, and Akt can block the activity of metabolic inhibitory kinases such as AMP-activated protein kinase (AMPK) and upregulate the metabolic activity of cells. It also inactivates cell cycle kinase inhibitors (p21, P27, etc.), thereby promoting cell growth, survival, and tumor-formation [33, 34]. MAPK8 (mitogen-activated protein kinase 8) gene, also known as JNK1/SAPK1 gene, is an important signal molecule in the MAPK signal transduction pathway, whose functions involve various mechanisms such as cell proliferation, cell differentiation, and apoptosis [35]. TNF*α* can promote adipocyte apoptosis, inhibit adipocyte differentiation, promote mature adipocyte dedifferentiation, promote intracellular lipolysis, and so on [36]. MAPK8 gene is an important signaling molecule in the above pathway of TNF*α*, which not only acts as an essential gene in the apoptosis mechanism but also initiates the death signaling pathway and apoptosis by inducing mitochondrial cytochrome C release [37]. All of these four hub genes are associated with the biosynthesis of fatty acid, maintaining cell growth, maintaining cell membrane stability, and regulating the conduction of receptor signals and other inflammatory reactions by affecting the functions of immune cells and inflammatory cells.

In summary, lipid metabolism is many complex and important biochemical reactions in the body and the basis of maintaining homeostasis, and ionizing radiation can cause metabolism of lipids in the blood and other tissues and organs and their substance and abnormality of lipid caused by abnormal accumulation, enhance the pathophysiological changes in oxidative stress, and affect the level of lipid and lipid metabolism imbalance. In our experiment, it was found that there were obvious lipid metabolism disorders in radiation-damaged mice, among which the most critical ones were linoleic acid metabolism, sphingolipid metabolism, and glycerolipid metabolism. G-Re can effectively regulate the metabolic level.

### 4.5. Glutathione Metabolism

Oxidative stress is a state in which excessive reactive oxygen species are produced in the body or the antioxidant defense function is weakened, and the balance between oxidation and antioxidant is damaged, resulting in tissue or cell damage [38]. Glutathione plays an important role in maintaining normal physiological activities of cells and is an important bioactive substance, antioxidant, and reducing buffer in the human body. It can activate the REDOX system and activate mercapto enzyme, thereby effectively removing free radicals and other reactive oxygen species and promoting carbohydrate metabolism, fat metabolism, and protein metabolism, thus effectively regulating cell membrane metabolism [39]. Glutathione comes in two forms, reduced glutathione (GSH) and oxidized glutathione (GSSG). However, GSSG can only act as reduction and oxidation when it is converted into reduced GSH under the action of nicotinamide adenine dinucleotide phosphate (NADPH) and GSR [40]. In our experiment, it was found that the content of GSSG decreased after radiation, while G-RE could effectively increase the activity of GSSG and reduce the level of oxidative stress.

## 5. Conclusion

In this study, a new comprehensive strategy was used to explore the key targets and mechanisms of Ginsenoside Re in the treatment of radiation damage based on the combination of metabolomics and network pharmacology. Pharmacodynamic analysis shows that G-Re can improve the thymus and spleen index, bone marrow DNA, and cytokine levels of radiation-damaged mice, reduce oxidative stress, and protect their hematopoietic system. Based on multivariate statistical analysis, a total of 16 potential biomarkers were identified in serum samples. These biomarkers are closely related to the occurrence and development of radiation damage. In addition, analysis of metabolic pathways showed that glycerophospholipid metabolism, linoleic acid metabolism, and sphingolipid metabolism were regulated in radiation injury model mice after G-Re intervention. Comprehensive network pharmacology and metabolomic analysis revealed 4 key targets and related metabolites and pathways, which were further validated by molecular docking. In conclusion, this study clarified the efficacy and potential mechanism of G-Re in improving radiation damage from the perspective of metabolomics and provided a method reference for analyzing the potential therapeutic effects and pharmacological mechanisms of TCM.

Although metabolomics and traditional Chinese medicine have been cross-studied, there are still many thought-provoking questions. Due to the existing analysis, technology is not perfect. It is impossible to completely analyze all the markers and trace metabolites of metabolic pathways in the organism. Secondly, according to the data analysis results of metabolomics, several differential metabolites and related metabolic pathways are selected to summarize the whole. However, whether the selected metabolites are appropriate and whether the metabolic stages of the intercepted pathways are comprehensive remain to be discussed. Our future research needs to focus on some differential metabolites screened, such as oxidized glutathione, linoleic acid, and palmitic acid, to further validate biomarkers and conduct quantitative analysis to determine the correlation between their amounts and radiation effects at different stages. It also needs to be integrated with multiomics to make metabolomics research more clear and comprehensive to study the mechanism of radiation damage.

## Figures and Tables

**Table 1:** Changes of G-Re on bone marrow DNA and spleen nodules in X-ray irradiated mice ($\bar{x}$ ± *s*, *n* = 10).

Group	Bone marrow DNA (*μ*g)	CFU-S
NC	346.60 ± 30.39	0
M	298.55 ± 17.37^*∗∗*^	4.909 ± 1.700
G-Re	328.73 ± 16.95^##^	6.909 ± 1.868^#^

Significant difference compared with control group, ^*∗*^*P* < 0.05, ^*∗∗*^*P* < 0.01; significant difference compared with model group, ^#^*P* < 0.05, ^##^*P* < 0.01.

**Table 2:** Effects of G-Re on serum IL-2, IL-6, IL-12, and IgM in irradiated mice (‾x ± s,n = 10).

Group	IL-2 (pg/mL)	IL-6 (pg/mL)	IL-12 (pg/mL)	IgM (*μ*g/mL)
NC	13.957 ± 1.245	13.897 ± 0.880	70.887 ± 9.250	0.623 ± 0.050
M	11.726 ± 1.539^*∗∗*^	15.315 ± 1.111^*∗∗*^	61.623 ± 6.368^*∗*^	0.562 ± 0.056^*∗*^
G-Re	13.769 ± 1.358^#^	14.885 ± 1.057^#^	66.500 ± 3.143^#^	0.612 ± 0.042^#^

Significant difference compared with control group, ^*∗*^*P* < 0.05, ^*∗∗*^*P* < 0.01; significant difference compared with model group, ^#^*P* < 0.05, ^##^*P* < 0.01.

**Table 3:** Potential biomarker information.

No.	ESI mode	HMDBID	Metabolites	Chemical formula	VIP	RT (min)	m/*z*	Normal vs model trend	Model vs G-Re trend
1	−	HMDB0009156	PE (18 : 3 (9Z, 12Z, 15Z)/18 : 0)	C_41_H_76_NO_8_P	3.8777	1.954	740.50656	Up	Down
2	−	HMDB0008429	PC (20 : 4 (5Z, 8Z, 11Z, 14Z)/16 : 0)	C_44_H_80_NO_8_P	2.5692	8.974	826.57028	Down	Up
3	−	HMDB0001085	Leukotriene B4	C_20_H_32_O_4_	2.6425	3.290	671.46118	Up	Down
4	−	HMDB0003337	Oxidized glutathione	C_20_H_32_N_6_O_12_S_2_	1.4469	3.467	593.13535	Down	Up
5	−	HMDB0002710	Prostaglandin J2	C_20_H_30_O_4_	3.1346	3.247	379.21586	Down	—
6	−	HMDB0000673	Linoleic acid	C_18_H_32_O_2_	4.7441	12.039	279.21364	Down	Up
7	+	HMDB0010384	LysoPC (18 : 0)	C_26_H_54_NO_7_P	3.2924	6.363	524.37289	Up	Down
8	+	HMDB0010385	LysoPC (18 : 1 (11Z))	C_26_H_52_NO_7_P	1.3872	5.511	522.35657	Up	Down
9	+	HMDB0010383	LysoPC (16 : 1 (9Z))	C_24_H_48_NO_7_P	1.4353	4.513	494.32409	Up	Down
10	+	HMDB0007981	PC (16 : 0/20 : 3 (8Z, 11Z, 14Z))	C_44_H_82_NO_8_P	6.2389	6.158	806.60484	Down	Up
11	+	HMDB0012096	SM (d18 : 1/12 : 0)	C_35_H_71_N_2_O_6_P	1.1461	7.789	669.49175	Up	Down
12	+	HMDB0000220	Palmitic acid	C_16_H_32_O_2_	8.4544	3.243	274.27847	Up	Down
13	+	HMDB0004610	Phytosphingosine	C_18_H_39_NO_3_	4.6911	3.272	318.30280	Down	Up
14	+	HMDB0000638	Dodecanoic acid	C_12_H_24_O_2_	2.6015	2.273	218.21566	Down	–
15	+	HMDB0000269	Sphinganine	C_18_H_39_NO_2_	1.7346	3.805	302.30810	Down	Up
16	+	HMDB0000806	Myristic acid	C_14_H_28_O_2_	2.6476	2.773	246.24757	Down	Up

**Table 4:** Metabolic pathways associated with potential biomarkers in serum.

Metabolic pathway	Total	Expected	Hits	Raw *p*	Holm adjust	FDR	Impact
Sphingolipid metabolism	21	0.18	3	0.00056	0.04726	0.0269	0.158
Linoleic acid metabolism	5	0.04	2	0.00064	0.05317	0.0269	1.0
Glycerophospholipid metabolism	36	0.30	3	0.00281	0.23015	0.0786	0.216
Fatty acid biosynthesis	47	0.39	3	0.00604	0.48925	0.1268	0.015
Biosynthesis of unsaturated fatty acids	36	0.30	2	0.03484	1.0	0.4878	0.0
Arachidonic acid metabolism	36	0.30	2	0.03484	1.0	0.4878	0.020
Alpha-linolenic acid metabolism	13	0.11	1	0.10409	1.0	1.0	0.0
Glycosylphosphatidylinositol (GPI)-anchor biosynthesis	14	0.12	1	0.11167	1.0	1.0	0.004
Glutathione metabolism	28	0.23	1	0.21173	1.0	1.0	0.027
Fatty acid elongation	39	0.33	1	0.28293	1.0	1.0	0.0
Fatty acid degradation	39	0.33	1	0.28293	1.0	1.0	0.0

**Table 5:** Target information of G-Re in the treatment of IR in network pharmacology.

No.	Gene symbol	Uniprot ID	Protein name
1	*STAT3*	P40763	Signal transducer and activator of transcription 3
2	*IL2*	P60568	Interleukin-2
3	*AKT2*	P31751	Serine/threonine-protein kinase AKT2
4	*ROCK1*	Q13464	Rho-associated protein kinase 1
5	*AKT1*	P31749	Serine/threonine-protein kinase AKT
6	*S1PR1*	P21453	Sphingosine 1-phosphate receptor Edg-1
7	*IGF1R*	P08069	Insulin-like growth factor I receptor
8	*EGFR*	P00533	Epidermal growth factor receptor erbB1
9	*MMP1*	P03956	Matrix metalloproteinase 1
10	*MAPK8*	P45983	Mitogen-activated protein kinase 8
11	*MAPK9*	P45984	Mitogen-activated protein kinase 9
12	*SYK*	P43405	Tyrosine-protein kinase SYK
13	*GRB2*	P62993	Growth factor receptor-bound protein 2
14	*MTOR*	P42345	Serine/threonine-protein kinase mTOR
15	*DPP4*	P27487	Dipeptidyl peptidase IV
16	*SLC37A4*	O43826	Glucose-6-phosphate translocase
17	*LTB4R*	Q15722	Leukotriene B4 receptor 1
18	*PIK3CA*	P42336	PI3-kinase p110-alpha subunit
19	*CSF1R*	P07333	Macrophage colony stimulating factor receptor
20	*ADRB2*	P07550	Adrenergic receptor beta
21	*NTRK1*	P04629	Nerve growth factor receptor Trk-A
22	*NTRK2*	Q16620	Neurotrophic tyrosine kinase receptor type 2
23	*ROCK2*	O75116	Rho-associated protein kinase 2
24	*HDAC6*	Q9UBN7	Histone deacetylase 6
25	*MMP9*	P14780	Matrix metalloproteinase 9
26	*MMP2*	P08253	Matrix metalloproteinase 2
27	*CDK2*	P24941	Cyclin-dependent kinase 2
28	*SELL*	P14151	Leukocyte adhesion molecule-1
29	*SELP*	P16109	P-selectin
30	*SRC*	P12931	Src tyrosine kinase
31	*GSR*	P00390	Glutathione reductase
32	*SOD2*	P04179	Superoxide dismutase
33	*PPP5C*	P53041	Serine/threonine-protein phosphatase 5
34	*PLA2G2A*	P14555	Phospholipase A2

**Table 6:** Information on key target genes, metabolites, and metabolic pathways.

Related pathways	Related target gene	Related metabolites
Linoleic acid metabolism	PLA2G2A	Linoleic acid
Fatty acid biosynthesis	PPP5, ROCK1, ROCK2, AKT1, AKT2, CDK2, MAPK8, EGFR, IGF1R, CSF1R, NTRK2, NTRK1, SRC, SYK	Palmitic acid, dodecanoic acid, myristic acid
Glutathione metabolism	GSR	Oxidized glutathione

## Data Availability

The data used to support the findings of this study are included within the article.

## Abbreviations

IR: Ionizing radiation
TCM: Traditional Chinese medicine
G-Re: Ginsenoside Re
ROS: Reactive oxygen species
CFU-S: Colony forming unit-spleen
HMDB: Human Metabolome Database
KEGG: Kyoto Encyclopedia of Genes and Genomes
VIP: Variable importance projection
PPI: protein interaction
PCA: Principal component analysis
PLS-DA: Partial least squares discriminant analysis
LysoPC: Lysophatidylcholine
SRC: Src tyrosine kinase
EGFR: Epidermal growth factor receptor
AKT: Serine/threonine-protein kinase
MAPK8: Mitogen-activated protein kinase 8
PC: Phosphatidylcholines
PE: Phosphatidylethanolamine.
